# Lesser Trochanter Apophyseal Avulsion Fracture: A Case Report and Review of the Literature

**DOI:** 10.7759/cureus.63236

**Published:** 2024-06-26

**Authors:** Christoopher Schwab, Rolando Zamora Jr, Samuel K Simister, Sarah Eldin, Zachary C Lum

**Affiliations:** 1 Department of Orthopedics, Nova Southeastern University, College of Osteopathic Medicine, Fort Lauderdale, USA; 2 Department of Emergency Medicine, Memorial Healthcare System, Fort Lauderdale, USA; 3 Department of Orthopedics, University of California Davis, Sacramento, USA

**Keywords:** apophyseal avulsion fracture, general orthopaedics, lesser trochanter avulsion injuries, orthopedic sports medicine, pediatric sports injury

## Abstract

Pediatric and adolescent apophyseal avulsion injuries are rare but important to recognize. This case report presents a 15-year-old male sprinter with a lesser trochanter apophyseal avulsion fracture who was treated nonoperatively. After 12 months of follow-up, conservative management resulted in significant healing and consolidation, with no pain or functional limitations at final follow-up. Conservative management of apophyseal avulsion injuries leads to positive outcomes in adolescents, while surgical intervention may be necessary for certain cases. Precise diagnosis and management are essential for successful outcomes, and in adults, a more extensive workup is recommended to rule out underlying malignancy.

## Introduction

Pediatric and adolescent apophyseal avulsion injuries, although uncommon, are important to examine closely to correctly obtain the diagnosis to guide management and clarify prognosis. The diagnosis of these fractures can be challenging as they often mimic other musculoskeletal conditions and have the potential to be overlooked in radiographic evaluations. Most avulsion fractures of the lower extremity occur at the ischial tuberosity, anterior superior iliac spine (ASIS), and anterior inferior iliac spine (AIIS); however, there is a paucity of literature reporting on the nonoperative management and functional outcomes.

Among these, lesser trochanteric apophyseal avulsion fractures are rare and make up less than 1% of all reported apophyseal avulsion fractures, typically occurring in young athletes during activities that involve abrupt, forceful movements [1]. The relevant anatomy for this injury involves the iliopsoas muscle, a powerful hip flexor originating from the bodies of the T12 to L5 vertebrae (psoas muscle) and the iliac fossa of the pelvis (iliacus muscle), combining to insert on the lesser trochanter of the femur [2].

Here, we present a case report and review current evidence in the literature on the management and outcomes of lesser trochanter apophyseal avulsion fractures in adolescents, supported by informed consent from the patient and guardians for publication of this report.

## Case presentation

**Figure 1:**
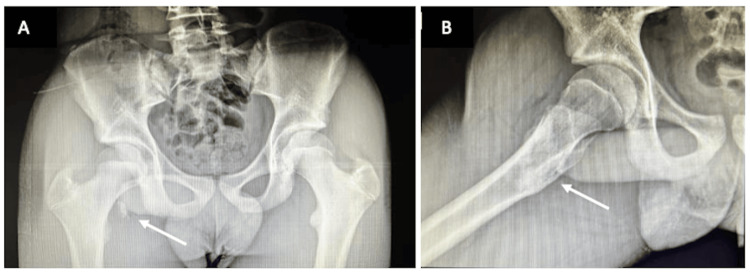
AP (a) and lateral (b) imaging of a lesser trochanter apophyseal avulsion fracture (arrows) in a 15-year-old male sprinter at the time of presentation AP: Anteroposterior

A 15-year-old male sprinter was running at his local high school during practice and felt a pop in his hip. He immediately noticed weakness with hip flexion, leading to an inability to sprint. The following day, he noticed groin pain and continued weakness. He presented to the orthopedic clinic one week later with continued complaints of hip pain, weakness, and inability to run. His pain by this time had improved, controlled with over-the-counter nonsteroidal anti-inflammatory drugs (NSAIDs), and he was able to walk with minimal pain. On physical exam, he had very mild tenderness over the AIIS, mild pain with resisted hip flexion, and very slight weakness, but overall had improved significantly from the initial injury. He had no pain with passive motion of the hip, internal/external rotation, nor tenderness over the ASIS. Radiographs showed a 1.5 cm proximally displaced lesser trochanter apophyseal avulsion fracture (Figure 1).

**Figure 2:**
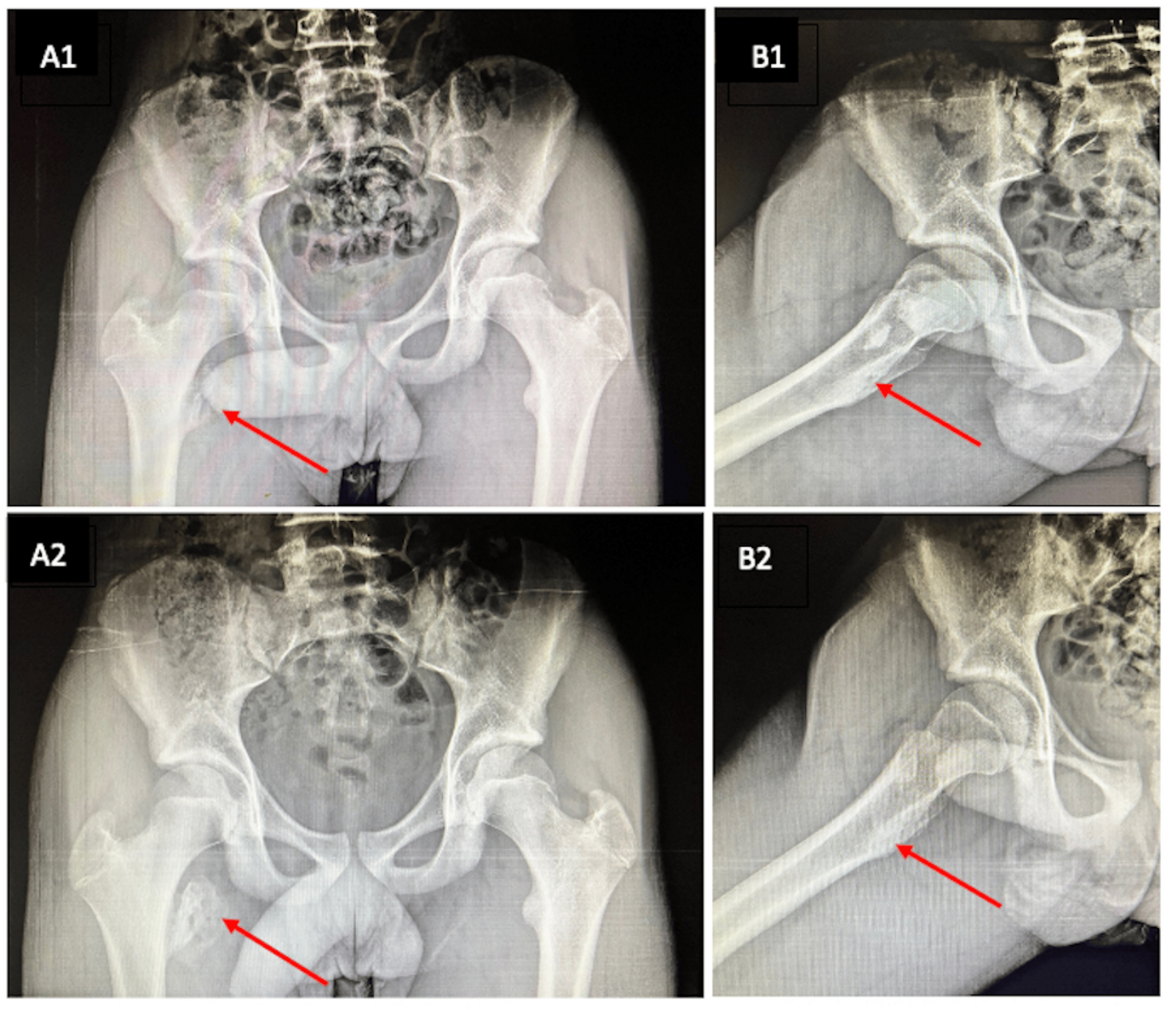
AP (A) and lateral (B) imaging showing healing of the lesser trochanter apophyseal avulsion fracture at three (1) and 12 months (2). Arrows indicating location of the lesser trochanter AP: Anteroposterior

Discussion with the family included the rare occurrences of these cases, with both operative and nonoperative risks, benefits, complications, and alternatives described. The patient was not planning to pursue elite level sports, and both pain and function was improving dramatically. It was decided to pursue nonoperative management, with non-weight bearing, and repeat radiographs in one month. Upon return, the patient had minimal pain and minimal functional limitations. His radiographs revealed significant bony healing and callus formation with radiographic concern for ilioischial impingement, but no clinical impingement could be detected. At final follow-up at three-months and one-year, radiographs had revealed consolidation of the lesser trochanter avulsion to a bony mass (Figure 2). The patient had no pain or functional limitations. There was no significant impediment in sports performance per his patient reported outcome measures (Table 1).

**Table 1:** Comparison of outcome measures in the present case VAS: Visual analogue scale

	Pre-injury	Injury	Post-injury
VAS pain scale	0	8	1
Harris hip score	100	28	93
Return to sport	-	-	9 months

## Discussion

Lesser trochanteric avulsion fractures in adolescents and children make up less than 1% of all reported apophyseal injury cases and sports medicine cases, and as a result, few case reports have been published regarding this topic. Data suggests that this injury occurs predominantly in male adolescents aged 13-15 before the lesser trochanter apophysis is fused at age 18 [1,2-7]. Avulsion fractures of the lesser trochanter can also occur in the adult population, typically in those greater than age 40; however, unlike in the pediatric population, this type of injury in adults is commonly due to an underlying malignancy [2]. The most common occurrence of these fractures in adolescents is during activities of sport when there is forceful contraction of the iliopsoas muscle, typically when either jumping or running [1,4-7].

**Figure 3:**
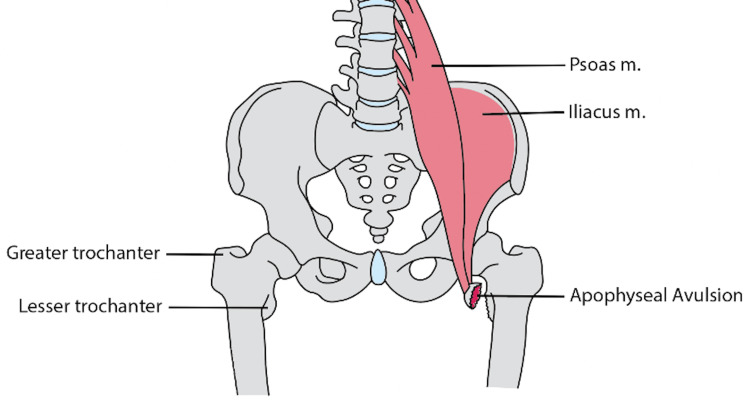
Anatomy of a lesser trochanter apophyseal avulsion Illustration by SK Simister

Workup for lesser trochanteric avulsion fractures can be difficult due to their rarity and the nonspecific symptoms based on anatomical location (see Figure 3). Commonly, the patient is an athlete who was running and/or jumping at the time of injury and will be unable to bear full weight. A physical exam will elicit pain in the groin and with hip flexion. The Ludloff test can be performed to examine if the patient can flex the hip in a sitting position with the knee fully extended or if there is pain while performing the test, examining the iliopsoas tendon. Naturally, radiographs can confirm the diagnosis and classify the amount of displacement. Utilizing other imaging modalities such as computerized tomography (CT) and magnetic resonance imaging (MRI) is typically not necessary, as it usually does not change the course of treatment. Vazquez et al. report that radiographs of nonossified bone may lead to false negatives, in which obtaining MRIs may be necessary [7].

Fractures that are classified as type I (nondisplaced), type II (<2 cm displacement), and type III (>2 cm displacement, no evidence of nonunion) are typically treated conservatively [5]. Nonoperative management includes rest, NSAIDs, physical therapy, and/or splinting with the goal of bearing full weight by six weeks post-injury. An open reduction, internal fixation should be considered when there is evidence of nonunion. Painful exostosis from impingement is another indication for surgical intervention, in which the exostosis should be excised, and the iliopsoas tendon reattached to the femur. In a previously published case series, all patients involved in the study underwent conservative treatment, and the average time to return to sport was approximately 11 weeks [2]. Athletes can return to pre-injury performance.

Since the lesser trochanter apophysis closes at 18 years of age, any avulsion fracture of the lesser trochanter in adults, especially those greater than 40 years of age, should warrant a more intense workup to rule out possible underlying malignancy [1]. If the fracture is traumatic in nature, treatment in an adult is typically conservative (rest, NSAIDs, physical therapy). Data has shown that nearly every traumatic fracture reported in adults was treated conservatively [1]. If a lesser trochanteric avulsion fracture is suspected in an adult with no history of trauma, imaging modalities, such as an MRI with contrast and CT chest/abdomen/pelvis, should be ordered to evaluate the location of the primary tumor site. In addition, a CT-guided biopsy should be performed. Tumor markers can help identify the origin of a possible malignancy, although care must be taken during interpretation since some of the markers are nonspecific. If a malignancy is found, patients may undergo a tumor resection and subsequent tumor prosthesis with intramedullary nailing, along with chemotherapy and radiation. Initial patient management for a malignancy diagnosis includes a multidisciplinary approach with medicine, oncology, interventional radiology, and orthopedic oncology to coordinate and render optimized care for best practice outcomes.

## Conclusions

Lesser trochanteric avulsion fractures are a rare form of injury that demonstrate a bimodal-age distribution. In adolescents, conservative treatment methods discussed result in good outcomes with a return to sport within three months post-injury. Indications such as nonunion and painful exostosis can be subject to surgery. In adults, lesser trochanteric avulsion fractures should be more extensively worked up to evaluate for other underlying pathology.
